# Assembly of a Hybrid *Formica aquilonia* × *F. polyctena* Ant Genome From a Haploid Male

**DOI:** 10.1093/jhered/esac019

**Published:** 2022-04-08

**Authors:** Pierre Nouhaud, Jack Beresford, Jonna Kulmuni

**Affiliations:** Organismal & Evolutionary Biology Research Programme, University of Helsinki, Biocenter 3, Viikinkaari 1, 00790 Helsinki, Finland; Tvärminne Zoological Station, University of Helsinki, J. A. Palménin tie 260, 10900 Hanko, Finland; Organismal & Evolutionary Biology Research Programme, University of Helsinki, Biocenter 3, Viikinkaari 1, 00790 Helsinki, Finland; Tvärminne Zoological Station, University of Helsinki, J. A. Palménin tie 260, 10900 Hanko, Finland; Organismal & Evolutionary Biology Research Programme, University of Helsinki, Biocenter 3, Viikinkaari 1, 00790 Helsinki, Finland; Tvärminne Zoological Station, University of Helsinki, J. A. Palménin tie 260, 10900 Hanko, Finland

**Keywords:** haplodiploidy, Hymenoptera, genome annotation, genome assembly, PacBio sequencing, wood ant

## Abstract

*Formica* red wood ants are a keystone species of boreal forest ecosystems and an emerging model system in the study of speciation and hybridization. Here, we performed a standard DNA extraction from a single, field-collected *Formica aquilonia* × *Formica polyctena* haploid male and assembled its genome using ~60× of PacBio long reads. After polishing and contaminant removal, the final assembly was 272 Mb (4687 contigs, N50 = 1.16 Mb). Our reference genome contains 98.5% of the core Hymenopteran BUSCOs and was pseudo-scaffolded using the assembly of a related species, *F. selysi* (28 scaffolds, N50 = 8.49 Mb). Around one-third of the genome consists of repeats, and 17 426 gene models were annotated using both protein and RNAseq data (97.4% BUSCO completeness). This resource is of comparable quality to the few other single individual insect genomes assembled to date and paves the way to genomic studies of admixture in natural populations and comparative genomic approaches in *Formica* wood ants.

Despite their small size, red wood ants (*Formica rufa* species group, hereafter wood ants) are heavyweights of boreal ecosystems. These social insects build massive interconnected nest mounds forming supercolonies of several million individuals, covering up to 2 km^2^ (Stockan et al. 2016). Wood ants are considered keystone species which play a role in nutrient cycling (Frouz et al. 2016), predator–prey dynamics or plant growth (Robinson et al. 2016), to name a few.

Wood ant genomics have so far mostly focused on supercoloniality, which is an extreme form of sociality. The canonical ant colony is headed by a single queen (monogyny) and occupies a unique nest (monodomy). Supercolonies are composed of several nests (polydomy) connected via inter-nest movement, each nest containing dozens to hundreds of unrelated egg-laying queens (polygyny, Pamilo 1993; Helanterä 2022). In wood ants, this social polymorphism is governed by a supergene maintained across species which diverged 40 Mya (Purcell et al. 2014, 2021; Brelsford et al. 2020).

Wood ants have undergone recent radiation (Goropashnaya et al. 2012; Borowiec et al. 2021) and represent a promising system for the study of speciation and hybridization. This process is ubiquitous across living organisms and haplodiploids (organisms for which one sex is haploid and the other, diploid) such as wood ants can answer some key questions in admixture research which are difficult to study in diploid organisms (Nouhaud et al. 2020). The best-characterized case is the occurrence of natural hybrids between *F. aquilonia* and *F. polyctena* in Southern Finland. Two hybrid lineages coexist in a single population (Kulmuni et al. 2010), where introgression between lineages is sex specific but could be modulated by external factors (Kulmuni et al. 2020). This hybrid population is relatively young (estimated age <50 generations) and has evolved without any significant gene flow from either species since admixture (Nouhaud et al. 2022).

Currently, no high-quality reference genome is available for any species of the *F. rufa* group. Kulmuni et al. (2020) assembled a draft genome using poolseq data from a hybrid *F. aquilonia* × *F. polyctena* population (both species belonging to the *F. rufa* group), but the assembly is highly fragmented (>300k contigs, N50 < 2 kbp). At a broader phylogenetic scale, among Palaearctic *Formica* species, 2 genomes are available for *F. exsecta* (Dhaygude et al. 2019) and *F. selysi* (Brelsford et al. 2020). Both species diverged around 20 Mya from the *F. rufa* species group (Borowiec et al. 2021).

While PacBio DNA input requirements have for a long time hindered the individual sequencing of small organisms, a modified SMRTbell library construction protocol was recently used to build a reference genome from a single *Anopheles* mosquito (Kingan, Heaton, et al. 2019). Few other recent examples demonstrate that high-quality arthropod genomes can now be built from a single individual (lanternfly: Kingan, Urban, et al. 2019; fruit fly: Adams et al. 2020; braconid wasp: Ye et al. 2020). Here, we assemble the genome of a single haploid, hybrid *F. aquilonia* × *F. polyctena* male using PacBio sequencing. As sexuals from these species are relatively big (~20 mg), we could apply a cost-effective, standard extraction protocol to obtain high-molecular-weight DNA from a single individual. The contigs were anchored against the *F. selysi* chromosomal assembly after contamination removal, and the genome was annotated using both RNAseq and protein data. Overall, the contiguity (N50 = 1.16 Mb) and completeness (98.5%) of the pseudo-scaffolded assembly are on par with other single individual arthropod genomes published to date, as well as other sequenced insect genomes (over 601 insect genomes analyzed by Hotaling et al. (2021), average N50 = 1.09 Mb; average completeness = 87.5%).

## Methods

### Biological Materials

All individuals used in the present study were sampled from the Långholmen population in Southern Finland (59°50ʹ59.9ʹʹN, 23°15ʹ03.3ʹʹE) in Spring 2018. This population has been characterized as a hybrid between *F. aquilonia* and *F. polyctena* using both genetic markers and morphological data (Kulmuni et al. 2010; Seifert et al. 2010). The Långholmen population is a supercolony consisting of 2 genetic lineages of hybrid origin (R and W [Kulmuni et al. 2010; Kulmuni and Pamilo 2014]), which show moderate genetic differentiation (*F*_ST_ ≈ 0.10, Kulmuni et al. 2020).

For long-read sequencing, a single haploid male was collected from the FAu2014a nest (W lineage) in Spring 2018. Sex determination was carried in the field using a morphological clue, the shape of the abdomen, which is long for males and round for females in both species. Results were then confirmed through the search of (duplicated) allelic contigs after assembly (see below). Two males and 2 unmated gynes (queens) from the same nest and lineage were also sampled at the same time for polishing purposes (short-read sequencing, see below). All samples were collected in individual sterile tubes and flash-frozen in the field. For RNA sequencing, sexual larvae were collected from multiple R and W nests in the same population in May 2014, measured, and put in individual tubes before flash-freezing in the laboratory within 24 h of collection (Beresford et al., unpublished data). All samples were stored at −80 ºC without any buffer.

### DNA Sequencing and Genome Assembly

#### Long-Read Sequencing

For both PacBio and Illumina DNA sequencing, all steps were carried out by Novogene (Hong Kong) as part of the Global Ant Genomics Alliance (GAGA, Boomsma et al. 2017). DNA from a single haploid male was extracted using a Sodium Dodecyl Sulfate (SDS) protocol following Pippel et al. (2020) and a SMRTbell library was prepared using the SMRT bell Template Prep Kit 1.0-SPv3 (Pacbio, 100-991-900). DNA quantification was performed using a Qubit fluorometer (Thermo Fisher) and purity was assessed with an agarose gel electrophoresis. The extraction from a single male yielded 9.89 μg of DNA, at a concentration of 86 ng/μL (A_260/280_ = 1.76, A_260/230_ = 1.20). DNA fragmentation was assessed through an Advanced Analytical Fragment Analyzer (AATI, mean size: 18 317 bp) prior to size selection (BluePippin, Sage Sciences, cutoff: 10 kb). The sample was loaded onto 4 SMRT cells with the Sequel Sequencing Kit 2.0 following PacBio recommendations and sequenced on a PacBio Sequel platform.

#### Short-Read DNA Sequencing

Since accuracy of long-read data is lower than short-read data (e.g., Koren et al. 2012; but see Wenger et al. 2019), Illumina data were generated to correct spurious base calls. For the 4 samples used for these polishing purposes, DNA was extracted from whole bodies with a SDS protocol and libraries were constructed using NEBNext DNA Library Prep Kits (New England Biolabs). Whole-genome sequencing was performed on Illumina Novaseq 6000 (paired-end mode, 150 bp), after which raw Illumina reads and adapter sequences were trimmed using Trimmomatic (v0.38; parameters LEADING:20 TRAILING:20 MINLEN:50; Bolger et al. 2014).

#### Whole-Genome Assembly

We assessed the performance of 2 long-read assemblers, Canu (v1.8, Koren et al. 2017) and wtdbg2 (v2.5, Ruan and Li 2020). We assumed a haploid genome size of 323 Mb, which is the mean size estimated from 5 species of the Formicinae subfamily by flow cytometry (Tsutsui et al. 2008). Canu was run with default parameters, except that the maximum allowed difference threshold was adapted to Sequel data (correctedErrorRate = 0.085), following Canu’s FAQ. For wtdbg2, a first run was performed using settings optimized for Sequel data and genome sizes below 1 Gb (preset 2: -p 0 -k 15 -AS 2 -s 0.05) but selecting all subread lengths (-L 0). Based on the subread distribution, a second run was performed with the same preset, but selecting only subreads above 10 kb (-L 10000). For each assembly, we assessed completeness using BUSCO (v4.0.5, Seppey et al. 2019) with the Hymenoptera ODB gene set v10.

The Canu assembly contained a total of 338 Mb in 3633 contigs (assuming a haploid genome size of 323 Mb, NG50 = 283 kb). The wtdbg2 assembly totaled 349 Mb in 11 615 contigs when using all subreads (wtdbg2-all, NG50 = 71 kb). Running wtdbg2 only with subreads greater than 10 kb (wtdbg2-10k, ~44×) yielded a 280 Mb assembly with 5098 contigs (NG50 = 689 kb). The wtdbg2-all assembly was discarded because it was missing a large fraction of BUSCOs (15.9%, Supplementary Table 1 online). The completeness of Canu and wtdbg2-10k assemblies were comparable (97.5% vs. 97.1%, respectively) despite stark differences in total sizes (338 Mb vs. 280 Mb, respectively). However, the BUSCO duplication rate was much higher for the Canu assembly (5.7% vs. 0.5%). This suggests that the Canu assembly may contain duplicated regions, which could in turn inflate its size. Interestingly, while the average genome size for the Formicinae subfamily was estimated at 323 Mb by flow cytometry (Tsutsui et al. 2008), recent genome projects within the *Formica* genus documented genome sizes of 278 Mb for *F. exsecta* (Dhaygude et al. 2019) and 290 Mb for *F. selysi* (Brelsford et al. 2020). Based on this observation, plus the assembly statistics and BUSCO score (Supplementary Table 1 online), we concluded that the wtdbg2-10k assembly was the best. The next steps were only performed on this assembly. Before polishing, we ran Purge Haplotigs (v1.1.1, Roach et al. 2018) to confirm that the individual sequenced was indeed haploid (see the unimodal distribution in Supplementary Figure 1 online).

### Assembly Polishing

To avoid incorporating sequencing errors in our final assembly (Watson and Warr 2019), we polished our contigs using Racon (v1.4.10, Vaser et al. 2017). We ran 4 polishing iterations with the PacBio data, followed by 2 iterations with the Illumina resequencing data (all 4 individuals pooled), always keeping unpolished sequences in the output (parameter -u). For each iteration, alignment was performed using minimap2 (v2.17, Li 2016, using parameters -x map-pb for PacBio and -ax sr for Illumina data, respectively). In our case, using data from different individuals, short-read polishing could be impacted by samples having different ancestries at a given locus. However, the same hybrid population (Långholmen) was sampled both for assembly and polishing purposes, and local ancestries are correlated across individuals within a hybrid population (Nouhaud et al. 2022): it is then unlikely that 2 ancestries still segregate at the same locus in the population.

### Contaminant Removal and Mitochondrial Genome Identification

The assembly was assessed for contaminants with BlobTools (v1.1.1, Laetsch and Blaxter 2017). Coverage files were obtained using minimap2 for both Canu-corrected PacBio subreads and the 4 resequenced individuals. Taxonomic partitioning of contigs was carried through BLAST against the NCBI non-redundant database. The contig containing the mitochondrial genome was identified based on BlobTools results (lower GC proportion compared to the rest of the genome and high sequencing depth, Supplementary Figure 2 online) and was further validated by BLAST of the *F. selysi* mtDNA sequence (Brelsford et al. 2020) against the whole assembly. *Formica* ants carry *Wolbachia* endosymbionts (Viljakainen et al. 2008) and horizontal gene transfer (HGT) has been previously characterized in *F. exsecta* (Dhaygude et al. 2019). To avoid classifying ant contigs impacted by HGT as contigs of endosymbiont origin, we blasted the closest *Wolbachia* genome (NCBI accession PRJNA436771) against our assembly and manually inspected these results in conjunction with coverage profiles and the physical location of Hymenoptera BUSCO hits (v4.0.5, Seppey et al. 2019).

### Pseudo-Scaffolding

Our polished, ant nuclear contigs were coalesced into pseudo-scaffolds with RaGOO (v1.1, Alonge et al. 2019), using the *F. selysi* reference genome (Brelsford et al. 2020) as a guide. To evaluate RaGOO’s performance, we also aligned contigs against *F. selysi* pseudo-chromosomes using the nucmer aligner from MUMmer (v4.0.0beta2, Marçais et al. 2018). Delta files from nucmer were processed using the DotPrep.py script (https://github.com/dnanexus/dot/blob/master/DotPrep.py, last accessed June 21, 2022) and alignments were visualized using Dot (https://dot.sandbox.bio/, last accessed June 21, 2022). A large portion (6 Mb) of Scaffold 10 in *F. selysi* mostly contained highly repetitive alignments (see also figure 1 from Brelsford et al. 2020). This region was removed from the *F. selysi* assembly before a second RaGOO run was performed. The gap size was set to 100 (100×N). All remaining, unanchored contigs were scaffolded as a single Scaffold 0. Of note, both parental species *F. aquilonia* and *F. polyctena* have 26 chromosomes (*n* = 26, Rosengren and Rosengren 1980), while *F. selysi* has 27 (*n* = 27). Our assembly contains 27 pseudo-scaffolds instead of the 26, which is the correct karyotype for both parental species. Combining available karyotype information (reviewed in Lorite and Palomeque 2010) to a recent phylogeny of wood ants (Borowiec et al. 2021) indicates that a chromosomal fusion occurred within the ancestor of the *F*. *rufa* group (*n* = 26) after its split with the lineage leading to *F. selysi* (*n* = 27).

### Annotation of Repeat Sequences

Transposable elements (TEs) were annotated using the Dfam TE Tools Container (v1.1, https://github.com/Dfam-consortium/TETools, last accessed October 20, 2020). A de novo consensus library was built with Repeatmodeler 2 (Flynn et al. 2020) and used to mask TE sequences in our assembly using Repeatmasker (Smit et al. 2013).

### RNA Sequencing

For annotation purposes, RNAseq data were generated for 9 individuals originating from 6 nests in the Långholmen population (R: nest FA4, 3 individuals and W: nest FA15, 1 individual; FA17, 2 individuals.; FA25, 1 individual; FA35, 1 individual; FAu2014a, 1 individual). These individuals were at different larval stages and total RNA was extracted from whole bodies using an ALLPrep DNA/RNA Mini Kit (Qiagen) following manufacturer’s instructions. Individual RNA qualities were assessed using a Bioanalyzer (Agilent 2100). Libraries were constructed using NEBNext Ultra RNA Library Prep Kits and samples were sequenced on an Illumina NextSeq platform (paired-end mode, 150 bp) at the Biomedicum Functional Genomics Unit (FuGU, University of Helsinki). Raw reads were trimmed using Trimmomatic (v0.38, parameters LEADING:20 TRAILING:20 SLIDINGWINDOW:5:20 MINLEN:50; Bolger et al. 2014) and unpaired reads were discarded. Approximately 5.60 million 150 bp paired reads were randomly sampled per individual and combined into 2 (Forward and Reverse) FASTQ files, totaling 50 million paired reads over all individuals.

### Genome Annotation

We annotated the genome with the Braker2 pipeline (v2.1.5, Hoff et al. 2019; Brůna et al. 2021). Both RNAseq- and protein-derived hints were used to train GeneMark-ETP, which predictions were in turn used to train Augustus and obtain the final gene set. All protein data available for Arthropoda were downloaded from OrthoDB (v10, Kriventseva et al. 2019, https://v100.orthodb.org/download/odb10_arthropoda_fasta.tar.gz, last accessed July 22, 2020) and aligned using ProtHint. This dataset contains 2.6 million sequences and encompasses 170 species, including 40 of the same order (Hymenoptera) and 17 of the same family (Formicidae). RNAseq data produced above were aligned against the hard-masked genome using STAR (v2.7.2, Dobin et al. 2013), and secondary alignments were removed with SAMtools (v1.10, Li et al. 2009). After the Braker2 run, protein sequences of all gene models not supported by at least one hint were blasted against the Uniprot database (UniProt Consortium 2019) and all models without any hit on Aculeata (wasps, bees, and ants) were discarded from the final gene set. Finally, functional annotation was carried out with EnTAP (v0.10.3, Hart et al. 2020) using the EggNOG (Huerta-Cepas et al. 2016), Uniprot, and RefSeq databases.

### Mapping-Based Evaluation

We compared our assembly and the *F. selysi* assembly (Brelsford et al. 2020, only chromosome-level assembly available for the Formica genus) by aligning short-read data from *F. aquilonia* and *F. polyctena* individuals sampled across Europe (*n* = 10 per species). These data were generated by Portinha et al. (2022) and we followed their trimming and mapping pipeline to align reads against the *F. selysi* assembly. Mapping statistics were then collected from BAM files using SAMtools.

## Results and Discussion

### Genome Sequencing and Assembly

We generated 2 547 044 subreads on the PacBio Sequel, summing up to 21.8 Gb of data (~68×). Half of the subreads were longer than 11.5 kb (NR50), with a mean length of 8.55 kb (Table 1).

**Table 1.** Software used for data analysis

Software	Version	Reference	Custom parameters (if any)
Canu	1.8	Koren et al. (2017)	correctedErrorRate=0.085
wtdbg2	2.5	Ruan and Li (2020)	-p 0 -k 15 -AS 2 -s 0.05 -L 10000
Busco	4.0.5	Seppey et al. (2019)	Hymenoptera ODB gene set v10
Purge Haplotigs	1.1.1	(Roach et al. 2018)	—
Racon	1.4.10	Vaser et al. (2017)	-u
Trimmomatic	0.38	Bolger et al. (2014)	LEADING:20 TRAILING:20 MINLEN:50
minimap2	2.17	Li (2016)	-x map-pb (PacBio)/ -ax sr (Illumina)
Blobtools	1.1.1	Laetsch and Blaxter (2017)	—
RaGOO	1.1	Alonge et al. (2019)	—
MUMmer	4.0.0beta2	Marçais et al. (2018)	—
Merqury	1.3	(Rhie et al. 2020)	—
Repeatmodeler2	2.0.1	Flynn et al. (2020)	-LTRStruct; via TETools container 1.1
Repeatmasker	4.1.0	(Smit et al. 2013)	via TETools container 1.1
Braker2	2.1.5	Bruna et al. (2020)	—
Star	2.7.2	Dobin et al. (2013)	—
SAMtools	1.10	Li et al. (2009)	—
EnTAP	0.10.3	Hart et al. (2020)	—

Running wtdbg2 only with subreads greater than 10 kb (~44×) yielded a 280 Mb assembly with 5098 contigs (assuming a haploid genome size of 323 Mb, NG50 = 689 kb, Supplementary Table 1 online). Interestingly, while the average genome size for the Formicinae subfamily was estimated at 323 Mb by flow cytometry (Tsutsui et al. 2008), recent genome projects within the *Formica* genus documented genome sizes much closer to our 280 Mb estimate, with 278 Mb for *F. exsecta* (Dhaygude et al. 2019) and 290 Mb for *F. selysi* (Brelsford et al. 2020). Based on this observation (similar assembly sizes for different *Formica* species) and BUSCO metrics, we concluded that our assembly had a sufficiently high level of completeness.

After polishing using both long (4 iterations) and short reads (2 iterations), the BUSCO score reached 98.5% for complete single-copy orthologs (Table 2) while the total size of the assembly reduced to 276 Mb.

**Table 2.** Assembly and annotation metrics

Genome assembly	
BUSCO v4.0.5 genome score	C: 98.5% [S: 97.9%, D: 0.6%], F: 0.4%, M: 1.1%, n: 5991
Number of contigs	4687
Contig N50 (bp)	1 163 114
Shortest contig (bp)	117
Longest contig (bp)	4 650 116
Average contig length (bp)	58 036
Total contig length (bp)	272 015 305
Number of pseudo-scaffolds	28
Pseudo-scaffold N50^a^ (bp)	8 490 488
Shortest pseudo-scaffold (bp)	3 646 393
Longest pseudo-scaffold^a^ (bp)	14 915 360
Average pseudo-scaffold length^a^ (bp)	7 887 222
Total pseudo-scaffold length (bp)	272 497 664
Total unanchored length (bp, fraction)	59 526 201 (21.8%)
GC content	36.3%
N fraction	0.17%
Genome annotation	
BUSCO v4.0.5 protein score	C: 97.4% [S: 96.8%, D: 0.6%], F: 1.4%, M: 1.2%, n: 5991
Total number of gene models	17 426
Mean gene length (bp)	5524
Average number of exons per gene	5.80
Number of models with RNAseq support (fraction)	11 956 (68.6%)
Number of isoforms	19 226
Average number of isoforms per gene	1.10
Cumulative gene length (bp, fraction)	78 835 002 (29.0%)
Cumulative exon length (bp, fraction)	27 442 032 (10.1%)
Repeat annotation	
Fraction of genome masked	32.01%
Interspersed repeats, total fraction	28.44%
Retroelements (class I)	6.39%
LINEs	1.47%
Gypsy/DIRS1	2.72%
DNA transposons (class II)	3.56%
Unclassified	18.50%
Simple repeats	2.59%

Scaffold statistics computed after excluding both the mitochondrial genome and Scaffold 0, which contains all unanchored contigs (59 Mb, “total unanchored length”).

Almost 92% (4688) of the 5098 contigs were assigned to Arthropoda, while 82 contigs were assigned to Proteobacteria (Supplementary Figure 2 online). *Formica* ants harbor *Wolbachia* endosymbionts (Viljakainen et al. 2008), and HGT between *Wolbachia* and the ant nuclear genome has been characterized (Dhaygude et al. 2019). Through manual curation, we assigned 76 contigs to *Wolbachia* (total size = 1 786 664 bp, N50 = 33.4 kb) and 6 contigs of the nuclear ant genome as putative HGTs. Overall, the contamination removal step decreased the nuclear ant genome size to 272 015 305 bp.

Finally, we anchored 78.2% (213 Mb, Table 2) of our assembly to the 27 pseudo-chromosomes of the *F. selysi* genome, a fraction similar to that of the original *F. selysi* study (78.3% of the assembly assigned to pseudo-chromosomes, see table S3 in Brelsford et al. 2020). The final QV score computed with Merqury (v1.3, Rhie et al. 2020) was 30.37 (error rate: 9.19 × 10^−4^).

**Figure 1.**
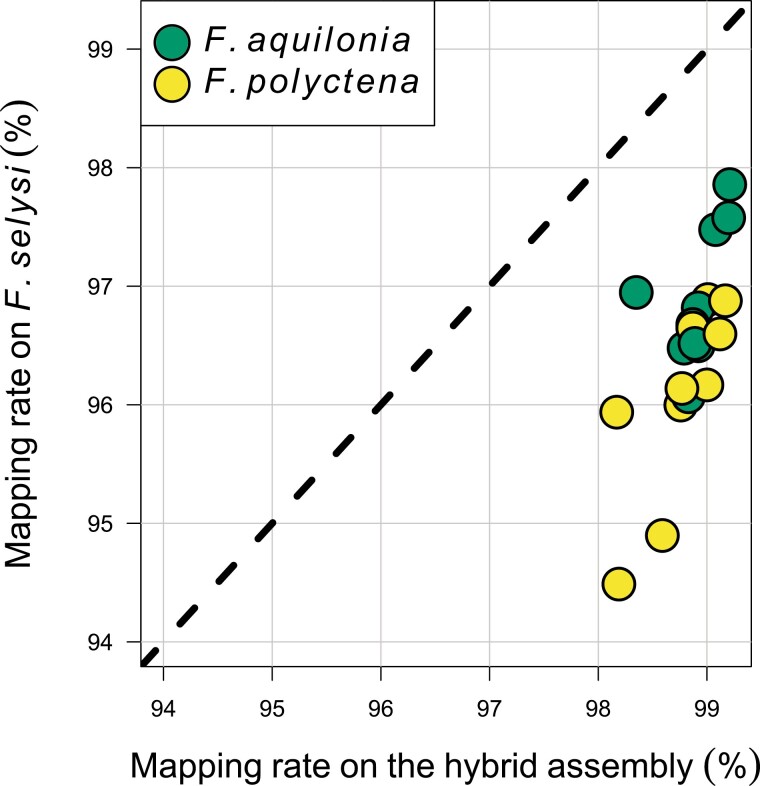
Comparison of mapping rates for *F. aquilonia* and *F. polyctena* individuals (*n* = 10 per species, data from Portinha et al. 2021) against our hybrid assembly (x-axis) and the *F. selysi* assembly (y-axis, Brelsford et al. 2020). The dashed line gives *y* = *x*.

The mean mapping rate of both *F. aquilonia* and *F. polyctena* individuals was slightly higher on our assembly compared to *F. selysi* (respectively 98.84% vs. 96.48%, Mann–Whitney test, W = 400, *P* = 6.78 × 10^−8^, Figure 1). This is expected since *F. selysi* diverged 5 Mya from the *F. rufa* species group, whereas species from the *F. rufa* species group (from which *F. aquilonia* and *F. polyctena* belong to) all diverged within the last 500 kya (Goropashnaya et al. 2012). This makes our assembly suitable for resequencing-based studies conducted on any of the 13 species of the *F. rufa* species group (e.g., Portinha et al. 2022).

### Genome Annotation

Overall, 32% of the sequence was masked with Repeatmasker, most of the repeats being unclassified (18.5%), 6.39% being retroelements and 3.56% being DNA transposons (Table 2). The vast majority of repeats were located on unanchored contigs (Supplementary Figure 3 online).

**Figure 2.**
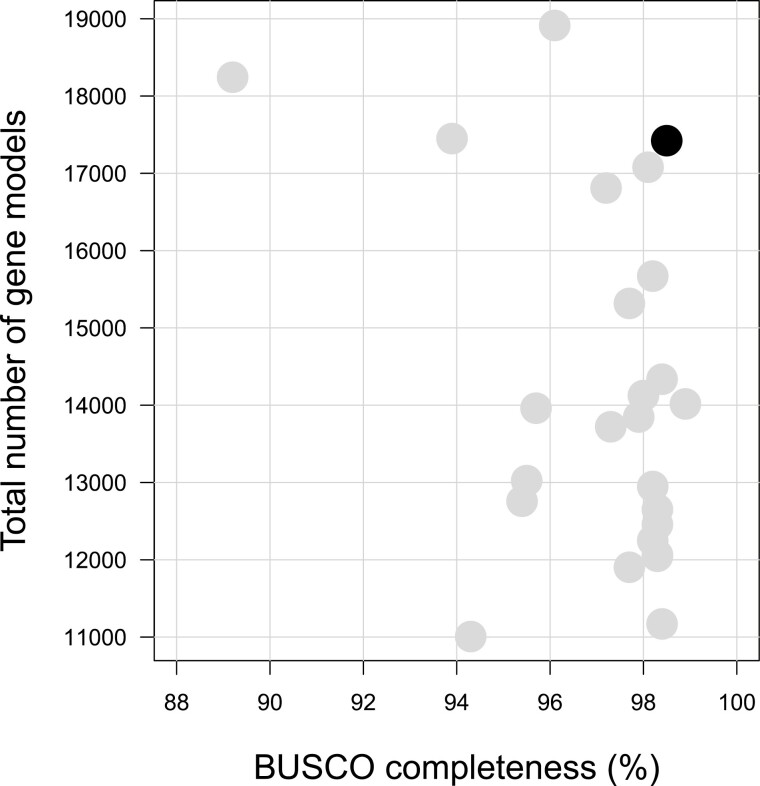
Total number of gene models as a function of BUSCO genome completeness metrics in ant genomes for which annotations are available on NCBI (*n* = 24, light gray) and the assembly of this study (black). Detailed statistics are shown in Supplementary Table 3 online.

The initial gene set contained 30 068 gene models, which is far superior to what has been documented in ants (~17 000 gene models, Gadau et al. 2012). Among these models, 14 287 (47.5%) were not supported by any protein or RNAseq hint. Moreover, the size of these hint-less models was much shorter than hint-supported models. As we suspected an overprediction problem (which was also observed for alternative Braker2 runs, Supplementary Table 2 online), we only kept hint-less models if their protein sequences had a blast hit against Aculeata in Uniprot, which reduced the total set from 30 068 to 17 426 gene models (15 781 with hints plus 1645 recovered after blast). Overall, 19 226 mRNAs were identified, among which 15 664 (81.5%) were functionally annotated with EnTAP. From these, 63.4% of the proteins had their best hit within ant species or *Drosophila melanogaster* (Supplementary Figure 4 online). The completeness of this final gene set assessed with BUSCO was good (protein mode: 97.4%, Table 2) and our assembly showed a level of completeness comparable to other ant genomes annotated so far (Figure 2, Supplementary Table 3 online).

## Conclusions

Here, we report the pseudo-scaffolded and annotated assembly of a single hybrid *F. aquilonia* × *F. polyctena* haploid male using a simple and cost-effective extraction protocol. The final assembly sums to 272 Mb, of which 78.2% are anchored onto 27 scaffolds, and recovers 98.5% of Hymenoptera-specific single-copy orthologs. Our annotation contains 17 426 protein-coding genes, with a BUSCO completeness of 97.4%.

Previously published single insect genomes have used either Nanopore or PacBio sequencing, sometimes coupled with whole-genome amplification or DNA extraction tailored to small starting material. We used standard extraction protocol from haploid tissue with PacBio sequencing and produced haploid reference genome reaching similar BUSCO and N50 statistics as previous single insect genomes (Supplementary Table 4 online).

This work provides a crucial resource to study speciation and contemporary hybridization, as well as the evolution of extreme sociality in the *F. rufa* species group, that contains 13 keystone species of forest ecosystems (Seifert 2021). The genome and its annotation are both of sufficient quality for studies aiming to reconstruct speciation histories (e.g., Portinha et al. 2022) and identify barrier loci or regions of adaptive introgression (e.g., Heliconius Genome Consortium 2012). It will also enable new approaches on the genomics of hybridization in this fascinating system (Nouhaud et al. 2020, 2022). Finally, it also demonstrates that high-quality arthropod genomes can be assembled from single individuals using standard, cost-effective protocols.

## Supplementary Material

esac019_suppl_Supplementary_Material

## Data Availability

The raw PacBio data and genome assembly have been deposited at the European Nucleotide Archive under the study PRJEB41943. The pseudo-scaffolded assembly, gene annotation, associated protein sequences, and RNAseq data used for annotation purposes are available on Figshare (doi: 10.6084/m9.figshare.c.5332442.v1 and 10.6084/m9.figshare.c.5277767).
