# Supplementary material for: Assembly of a Hybrid Formica aquilonia × F. polyctena Ant Genome From a Haploid Male
Source: J Hered. 2022 Apr 8;113(3):353–9. doi: 10.1093/jhered/esac019 (PMC9270870; doi:10.1093/jhered/esac019)
Supplement: esac019_suppl_Supplementary_Material [file esac019_suppl_supplementary_material.pdf]

Supplementary material for

Assembly of a hybrid *Formica aquilonia* × *F. polyctena* ant genome from a haploid male

Pierre Nouhaud [1,2,*], Jack Beresford [1,2] & Jonna Kulmuni [1,2]

[1] Organismal & Evolutionary Biology Research Programme, University of Helsinki, Helsinki, Finland
[2] Tvärminne Zoological Station, University of Helsinki, Hanko, Finland
* Corresponding author: Pierre Nouhaud, Organismal & Evolutionary Biology Research Programme, University of Helsinki, Helsinki, Finland, pierr3.nouhaud@gmail.com

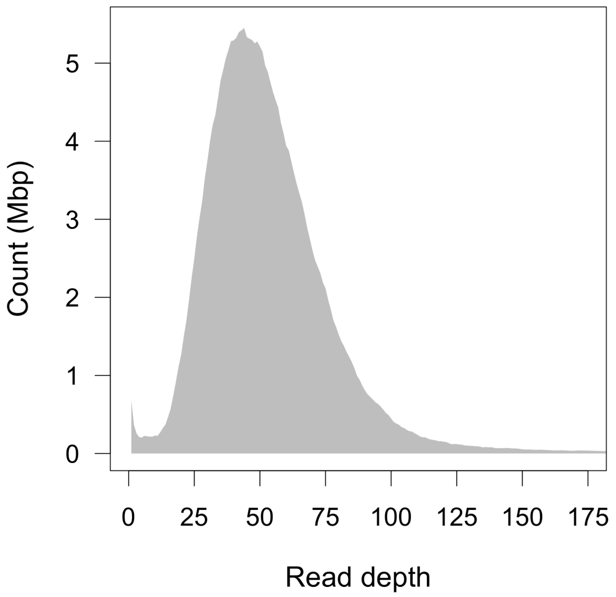

Supplementary figure 1. Genome-wide read-depth histogram computed with Purge Haplotigs for the wtdbg2-10k assembly. Unimodal distribution supports a lack of allelic contigs, as expected if the single male individual sequenced was indeed haploid.

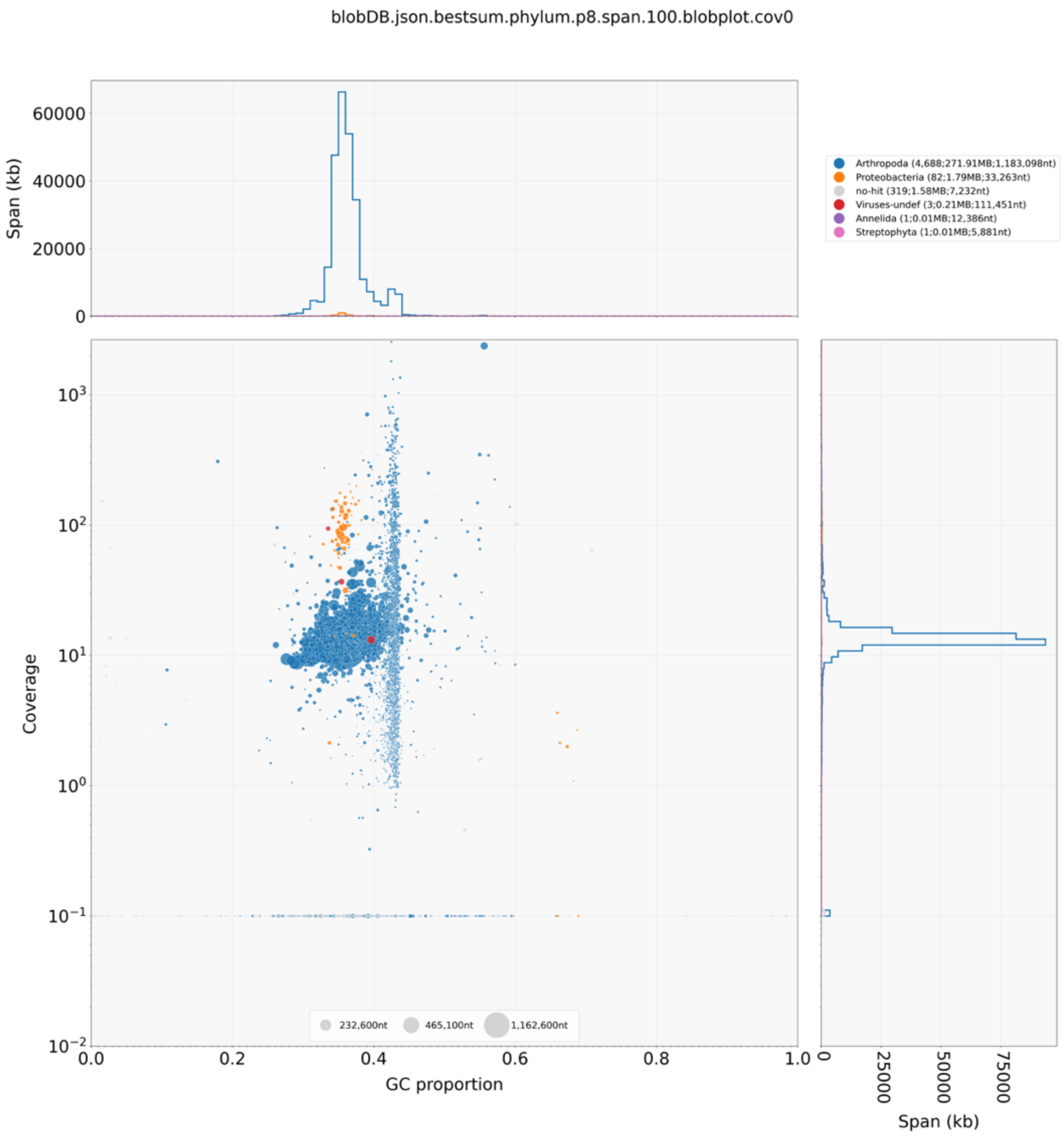

Supplementary figure 2. Results of the contamination assessment for the wtdbg2-10k assembly using Blobtools and the PacBio data.

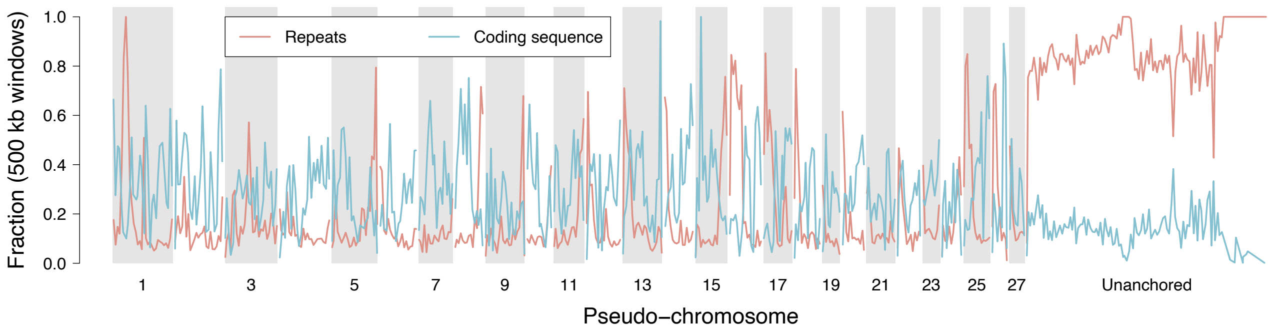

Supplementary figure 3. Fraction of repeats and coding sequences computed genome-wide in non-overlapping 500kb windows. Note that the unanchored portion of the genome is repeat-rich and gene-poor.

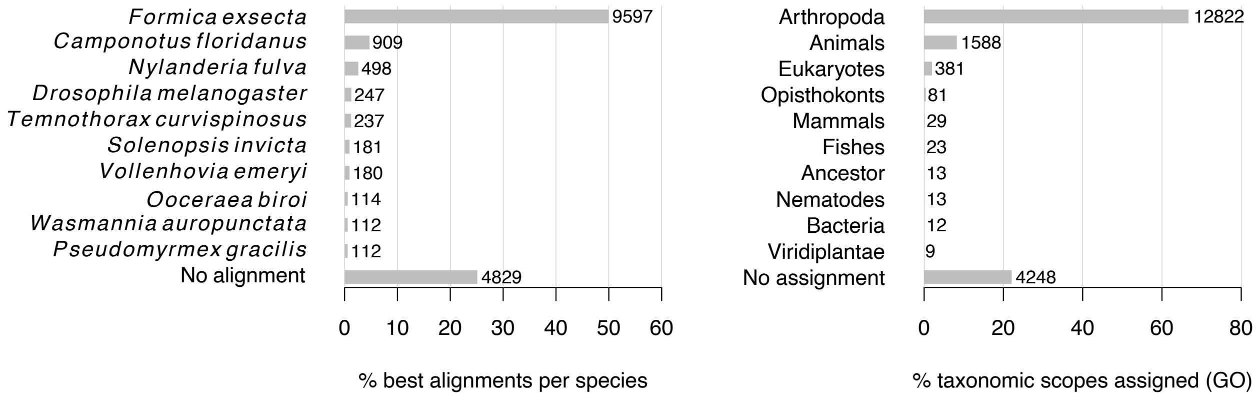

Supplementary figure 4. Results of the functional annotation pipeline. The left panel shows the first ten species with the highest number of best hits after similarity search using Diamond and both RefSeq nr and Uniprot databases. Apart from the fruit fly *D. melanogaster* (ranked 4th), all ten most abundant species are ants. Overall, 63.4% of the proteome has best hits within one of these species (25.1% did not get any proper alignment). On the right panel are displayed the number and fraction of transcripts assigned to the first ten Gene Ontology taxonomic scopes using EggNOG.

Supplementary table 1. Raw assembly statistics. All sizes are given in base pairs, and statistics are computed assuming a haploid genome size of 323 Mb (see main text).

Assembler	Subread size cutoff	Total assembly size	Number of contigs	Longest contig	N50	L50	N75	L75	NG50	LG50	NG75	LG75	BUSCO v4.0.5 genome score
canu	0 (none)	338082226	3633	3815783	260777	304	93570	832	260777	304	93947	831	C:97.5%[S:91.8%,D:5.7%],F:1.0%,M:1.5%,n:5991
wtdbg2	0 (none)	349682633	11615	781157	59862	1255	23343	3721	70590	1051	28457	2946	C:79.5%[S:79.1%,D:0.4%],F:4.6%,M:15.9%,n:5991
wtdbg2	10000	280368309	5098	4663969	1125534	68	191235	231	689459	93	57231	534	C:97.1%[S:96.6%,D:0.5%],F:1.3%,M:1.6%,n:5991

Supplementary table 2. Annotation statistics for alternative Braker runs. The etp run was considered the best based on the BUSCO score, and its output was later filtered to get rid of spurious gene models.

Braker2 mode	Data		Total number of gene models	BUSCO v4.0.5 genome score
	RNAseq	Protein		
ep	no	yes	32,500	C:95.6%[S:94.9%,D:0.7%],F:2.3%,M:2.1%,n:5991
et	yes	no	33,077	C:95.5%[S:94.8%,D:0.7%],F:2.0%,M:2.5%,n:5991
etp	yes	yes	30,068	C:97.4%[S:96.8%,D:0.6%],F:1.4%,M:1.2%,n:5991

Supplementary table 3. Assembly and annotation metrics for the 24 ant genomes for which annotations are available on NCBI.

Organism Scientific Name	Taxonomy id	Assembly Name	Assembly Accession	Source	Contig N50 (bp)	Size (bp)	Submission Date	BUSCO v4.0.5 genome score	Annotation	Gene Count	Reference
Lasius niger	67767	ASM104565v1	GCA_001045655.1	GenBank	17048	236236391	2.7.2015	C:89.2%[S:88.5%,D:0.7%], F:7.9%,M:2.9%,n:5991	INSDC submitter	18247	(Konorov et al. 2017)
Temnothorax longispinosus	300112	tlon_1.0	GCA_004794745.1	GenBank	30134	260681563	15.4.2019	C:95.5%[S:95.0%,D:0.5%], F:2.4%,M:2.1%,n:5991	INSDC submitter	13028	(Kaur et al. 2019)
Atta cephalotes	12957	Attacep1.0	GCF_000143395.1	RefSeq	14798	317671980	6.7.2010	C:94.3%[S:94.3%,D:0.0%], F:2.8%,M:2.9%,n:5991	NCBI Annotation Release 100	11011	(Suen et al. 2011)
Pogonomyrmex barbatus	144034	Pbar_UMD_V03	GCF_000187915.1	RefSeq	11605	235645958	4.2.2011	C:95.4%[S:95.3%,D:0.1%], F:2.6%,M:2.0%,n:5991	NCBI Annotation Release 101	12761	(C. R. Smith et al. 2011)
Solenopsis invicta	13686	Si_gnH	GCF_000188075.2	RefSeq	21162	398979080	1.8.2018	C:97.2%[S:96.9%,D:0.3%], F:1.4%,M:1.4%,n:5991	NCBI Annotation Release 103	16814	(Wurm et al. 2011)
Acromyrmex echinatior	103372	Aech_3.9	GCF_000204515.1	RefSeq	80630	295944863	3.5.2011	C:98.2%[S:97.9%,D:0.3%], F:0.8%,M:1.0%,n:5991	NCBI Annotation Release 100	12253	(Nygaard et al. 2011)
Linepithema humile	83485	Lhum_UMD_V04	GCF_000217595.1	RefSeq	35858	219500750	10.6.2011	C:98.2%[S:98.0%,D:0.2%], F:0.8%,M:1.0%,n:5991	NCBI Annotation Release 100	12952	(C. D. Smith et al. 2011)
Vollenhovia emeryi	411798	V.emery_V1.0	GCF_000949405.1	RefSeq	32417	287900827	6.3.2015	C:98.2%[S:97.8%,D:0.4%], F:1.0%,M:0.8%,n:5991	NCBI Annotation Release 100	15674	(C. R. Smith et al. 2015)
Wasmannia auropunctata	64793	wasmannia.A_1.0	GCF_000956235.1	RefSeq	37912	324120201	17.3.2015	C:97.7%[S:97.4%,D:0.3%], F:1.2%,M:1.1%,n:5991	NCBI Annotation Release 100	15321	Not published
Dinoponera quadriceps	609295	ASM131382v1	GCF_001313825.1	RefSeq	29911	259665865	13.10.2015	C:97.7%[S:97.5%,D:0.2%], F:1.1%,M:1.2%,n:5991	NCBI Annotation Release 100	11907	(Patalano et al. 2015)
Atta colombica	520822	Acol1.0	GCF_001594045.1	RefSeq	15290	291257934	25.3.2016	C:98.4%[S:98.2%,D:0.2%], F:0.8%,M:0.8%,n:5991	NCBI Annotation Release 100	11174	Not published
Trachymyrmex zeteki	64791	Tzet1.0	GCF_001594055.1	RefSeq	52131	267973152	31.3.2016	C:98.3%[S:98.2%,D:0.1%], F:0.8%,M:0.9%,n:5991	NCBI Annotation Release 100	12066	(Nygaard et al. 2016)
Cyphomyrmex costatus	456900	Ccosl1.0	GCF_001594065.1	RefSeq	74312	300316566	25.3.2016	C:98.3%[S:97.7%,D:0.6%], F:0.7%,M:1.0%,n:5991	NCBI Annotation Release 100	12460	Not published
Trachymyrmex cornetzi	471704	Tcor1.0	GCF_001594075.1	RefSeq	29356	369438293	25.3.2016	C:97.9%[S:97.6%,D:0.3%], F:1.1%,M:1.0%,n:5991	NCBI Annotation Release 100	13851	Not published
Trachymyrmex septentrionalis	34720	Tsep1.0	GCF_001594115.1	RefSeq	14962	291747019	25.3.2016	C:98.3%[S:98.1%,D:0.2%], F:0.7%,M:1.0%,n:5991	NCBI Annotation Release 100	12049	Not published
Pseudomyrmex gracilis	219809	ASM200609v1	GCF_002006095.1	RefSeq	38830	282776121	23.2.2017	C:98.3%[S:97.8%,D:0.5%], F:0.7%,M:1.0%,n:5991	NCBI Annotation Release 100	12655	(Rubin and Moreau 2016)
Temnothorax curvispinosus	300111	ASM307098v1	GCF_003070985.1	RefSeq	38942	303539295	24.4.2018	C:93.9%[S:88.1%,D:5.8%], F:2.4%,M:3.7%,n:5991	NCBI Annotation Release 100	17453	Not published
Harpegnathos saltator	610380	Hsal_v8.5	GCF_003227715.1	RefSeq	911506	334536844	14.6.2018	C:98.4%[S:97.6%,D:0.8%], F:0.7%,M:0.9%,n:5991	NCBI Annotation Release 102	14340	(Bonasio et al. 2010)
Camponotus floridanus	104421	Cflo_v7.5	GCF_003227725.1	RefSeq	1278439	284009182	14.6.2018	C:98.9%[S:98.4%,D:0.5%], F:0.4%,M:0.7%,n:5991	NCBI Annotation Release 102	14020	(Bonasio et al. 2010)
Formica exsecta	72781	ASM365146v1	GCF_003651465.1	RefSeq	24299	277633851	15.10.2018	C:97.3%[S:95.0%,D:2.3%], F:1.5%,M:1.2%,n:5991	NCBI Annotation Release 100	13725	(Dhaygude et al. 2019)
Ooceraea biroi	2015173	Obir_v5.4	GCF_003672135.1	RefSeq	3735272	223876465	23.10.2018	C:98.0%[S:97.5%,D:0.5%], F:0.7%,M:1.3%,n:5991	NCBI Annotation Release 100	14128	(Oxley et al. 2014)
Nylanderia fulva	613905	TAMU_Nfulva_1.0	GCF_005281655.1	RefSeq	320712	375107333	13.5.2019	C:96.1%[S:94.2%,D:1.9%], F:1.4%,M:2.5%,n:5991	NCBI Annotation Release 100	18917	Not published (i5k)
Odontomachus brunneus	486640	Obru_v1	GCF_010583005.1	RefSeq	22002	393036571	13.2.2020	C:95.7%[S:95.1%,D:0.6%], F:2.1%,M:2.2%,n:5991	NCBI Annotation Release 100	13965	Not published (i5k)
Monomorium pharaonis	307658	ASM1337386v2	GCF_013373865.1	RefSeq	1861574	325506644	14.8.2020	C:98.1%[S:95.9%,D:2.2%], F:0.9%,M:1.0%,n:5991	NCBI Annotation Release 102	17083	(Mikheyev and Linksvayer 2015)

Supplementary table S4. Comparison of some recent single-individual-based arthropod genome assemblies.

Study	Species	Extraction	Input amount	Sequencing Platform	Contig number	Assembly size (Mb)	N50 (Mb)	Scaffolding	Complete BUSCOs
Kingan, Heaton et al. 2020	mosquito *Anopheles coluzzii*	Modified Qiagen MagAttract protocol	100 ng	PacBio	206	251	3.47	No	98.00 %
Kingan, Urban et al. 2020	lanternfly *Lycorma delicatula*	Modified Chromium™ Genome Protocol	5 µg	PacBio	2927	2 252	1.52	No	96.80 %
Adams et al. 2020	fruit fly *Drosophila melanogaster*	Qiagen MagAttract	78.3 ng	Illumina, Nanopore, HiC	NA	111	26.3	Hi-C	95.20 %
Ye et al. 2020	parasitoid wasp *Habrobracon hebetor*	TIANamp Micro DNA Kit with WGA	20 ng	Nanopore	765	132	1.63	No	99.00 %
This study	wood ant *Formica aquilonia* × *F. polyctena* hybrid	standard SDS	9.89 µg	PacBio	4687	272	1.16	Reference-guided	98.50 %

References

Bonasio, Roberto, Guojie Zhang, Chaoyang Ye, Navdeep S. Mutti, Xiaodong Fang, Nan Qin, Greg Donahue, et al. 2010. "Genomic Comparison of the Ants Camponotus Floridanus and Harpegnathos Saltator." *Science* 329 (5995): 1068–71.

Dhaygude, Kishor, Abhilash Nair, Helena Johansson, Yannick Wurm, and Liselotte Sundström. 2019. "The First Draft Genomes of the Ant Formica Exsecta, and Its Wolbachia Endosymbiont Reveal Extensive Gene Transfer from Endosymbiont to Host." *BMC Genomics* 20 (1): 301.

Kaur, Rajbir, Marah Stoldt, Evelien Jongepier, Barbara Feldmeyer, Florian Menzel, Erich Bornberg-Bauer, and Susanne Foitzik. 2019. "Ant Behaviour and Brain Gene Expression of Defending Hosts Depend on the Ecological Success of the Intruding Social Parasite." *Philosophical Transactions of the Royal Society of London. Series B, Biological Sciences* 374 (1769): 20180192.

Konorov, Evgenii A., Mikhail A. Nikitin, Kirill V. Mikhailov, Sergey N. Lysenkov, Mikhail Belenky, Peter L. Chang, Sergey V. Nuzhdin, and Victoria A. Scobeyeva. 2017. "Genomic Exaptation Enables Lasius Niger Adaptation to Urban Environments." *BMC Evolutionary Biology* 17 (Suppl 1): 39.

Mikheyev, Alexander S., and Timothy A. Linksvayer. 2015. "Genes Associated with Ant Social Behavior Show Distinct Transcriptional and Evolutionary Patterns." *eLife* 4 (January): e04775.

Nygaard, Sanne, Haofu Hu, Cai Li, Morten Schiøtt, Zhensheng Chen, Zhikai Yang, Qiaolin Xie, et al. 2016. "Reciprocal Genomic Evolution in the Ant-Fungus Agricultural Symbiosis." *Nature Communications* 7 (July): 12233.

Nygaard, Sanne, Guojie Zhang, Morten Schiøtt, Cai Li, Yannick Wurm, Haofu Hu, Jiajian Zhou, et al. 2011. "The Genome of the Leaf-Cutting Ant Acromyrmex Echinatior Suggests Key Adaptations to Advanced Social Life and Fungus Farming." *Genome Research* 21 (8): 1339–48.

Oxley, Peter R., Lu Ji, Ingrid Fetter-Pruneda, Sean K. McKenzie, Cai Li, Haofu Hu, Guojie Zhang, and Daniel J. C. Kronauer. 2014. "The Genome of the Clonal Raider Ant Cerapachys Biroi." *Current Biology: CB* 24 (4): 451–58.

Patalano, Solenn, Anna Vlasova, Chris Wyatt, Philip Ewels, Francisco Camara, Pedro G. Ferreira, Claire L. Asher, et al. 2015. "Molecular Signatures of Plastic Phenotypes in Two Eusocial Insect Species with Simple Societies." *Proceedings of the National Academy of Sciences of the United States of America* 112 (45): 13970–75.

Rubin, Benjamin E. R., and Corrie S. Moreau. 2016. "Comparative Genomics Reveals Convergent Rates of Evolution in Ant-Plant Mutualisms." *Nature Communications* 7 (August): 12679.

Smith, Chris R., Sara Helms Cahan, Carsten Kemena, Seán G. Brady, Wei Yang, Erich Bornberg-Bauer, Ti Eriksson, et al. 2015. "How Do Genomes Create Novel Phenotypes? Insights from the Loss of the Worker Caste in Ant Social Parasites." *Molecular Biology and Evolution* 32 (11): 2919–31.

Smith, Chris R., Christopher D. Smith, Hugh M. Robertson, Martin Helmkampf, Aleksey Zimin, Mark Yandell, Carson Holt, et al. 2011. "Draft Genome of the Red Harvester Ant Pogonomyrmex Barbatus." *Proceedings of the National Academy of Sciences of the United States of America* 108 (14): 5667–72.

Smith, Christopher D., Aleksey Zimin, Carson Holt, Ehab Abouheif, Richard Benton, Elizabeth Cash, Vincent Croset, et al. 2011. "Draft Genome of the Globally Widespread and Invasive Argentine Ant (Linepithema Humile)." *Proceedings of the National Academy of Sciences of the United States of America* 108 (14): 5673–78.

Suen, Garret, Clotilde Teiling, Lewyn Li, Carson Holt, Ehab Abouheif, Erich Bornberg-Bauer, Pascal Bouffard, et al. 2011. "The Genome Sequence of the Leaf-Cutter Ant Atta Cephalotes Reveals Insights into Its Obligate Symbiotic Lifestyle." *PLoS Genetics* 7 (2): e1002007.

Wurm, Yannick, John Wang, Oksana Riba-Grognuz, Miguel Corona, Sanne Nygaard, Brendan G. Hunt, Krista K. Ingram, et al. 2011. "The Genome of the Fire Ant Solenopsis Invicta." *Proceedings of the National Academy of Sciences of the United States of America* 108 (14): 5679–84.
